# Surgical vs. non-surgical therapy for partial tears of the rotator cuff: a systematic review and meta-analysis of pooled studies with indirect comparison

**DOI:** 10.1186/s12891-026-09938-z

**Published:** 2026-05-18

**Authors:** Elena Ricker, Lara Stehling, Lisa Klute, Leopold Henßler, Helge Knüttel, Florian Zeman, Volker Alt, Maximilian Kerschbaum

**Affiliations:** 1 Clinic of Trauma Surgery, University Medical Center Regensburg, Franz-Josef-Strauss-Allee 11, D-93053 Regensburg, Germany; 2 Medical Branch Library, University Library, University of Regensburg, Universitätsstraße 31, 93053 Regensburg, Germany; 3 Center for Clinical Studies, University Medical Center Regensburg, Franz-Josef-Strauss-Allee 11, 93053 Regensburg, Germany

**Keywords:** Shoulder, Rotator Cuff, Partial Thickness Rotator Cuff Tear, Surgical Therapy, Non-Operative Management

## Abstract

**Background:**

Rotator cuff disease (RCD) constitutes the most common cause of shoulder pain, with partial-thickness rotator cuff tears (PT-RCTs) representing a substantial subset that may progress over time. Although both surgical and non-surgical interventions are employed in clinical practice, a consensus regarding the optimal management of symptomatic PT-RCTs is lacking, and a comprehensive synthesis of available evidence has not yet been conducted.

**Methods:**

A systematic review was conducted according to PRISMA guidelines using MEDLINE, EMBASE, CENTRAL, ClinicalTrials.gov, and WHO ICTRP. Studies on arthroscopic surgical and/or non-surgical treatments for PT-RCTs in patients ≥ 18 years were included. Clinical outcomes (Constant Score (CS), ASES Score, VAS for pain) were extracted. Random-effects meta-analysis and descriptive statistics were used to analyze outcomes and study characteristics.

**Results:**

Of the 9,894 trials screened, 33 trials with a total of 1,818 patients met the inclusion criteria. Comparing pooled weighted mean outcomes across studies, surgical cohorts demonstrated higher scores than non-surgical cohorts in both the CS (*p* = 0.0095) and the ASES (*p* = 0.0060). However, no specific surgical technique proved superior, with neither Reconstruction versus Debridement (CS, *p* = 0.19; ASES, *p* = 0.06) nor Tear Completion versus Transtendon Repair (CS, *p* = 0.13; ASES, *p* = 0.65) reaching statistical significance. Although surgical treatment was statistically superior to non-surgical approaches, the observed differences remained below the minimal clinically important differences (MCID), indicating limited clinical relevance.

**Conclusion:**

Pooled analyses showed higher Constant and ASES scores in surgical cohorts. However, the magnitude of this difference does not exceed MCID, indicating limited clinical relevance. No surgical technique demonstrated significant superiority. These findings highlight the need for individualized treatment decisions, considering the limited clinical benefit of surgery over non-surgical approaches.

**Protocol Registration:**

The protocol was registered in PROSPERO with ID: CRD42023487714

## Background

Shoulder pain is a prevalent condition, affecting 6.7% to 66.7% of the general population [1] representing the third most common musculoskeletal complaint in primary care [2]. Rotator cuff disorders (RCD) account for up to 85% of cases [3–5] and are expected to increase with an aging population further increasing the burden on healthcare systems and affected individuals [6]. The associated impact on quality of life is comparable to that of chronic conditions such as hypertension and diabetes [7]. 

RCD includes a spectrum of pathologies from tendinopathy to complete tendon tears [8]. Among these, partial-thickness rotator cuff tears (PT-RCTs) represent 17% to 37% [9] of cases and are approximately twice as prevalent as full-thickness rotator cuff tears (FT-RCTs) [10]. While some rotator cuff tears are the result of trauma, most are degenerative, with multifactorial causes including age-related tissue changes, reduced vascularity, impingement and glenohumeral instability [11]. PT-RCTs are prone to progression over time, potentially leading to full-thickness tears, greater functional impairment, and an increased need for medical intervention [10]. 

Treatment options are generally categorized as surgical or non-surgical [11]. This analysis focuses on comparing these two approaches. Surgical treatments, as defined in this study, include arthroscopic refixation (transtendinous repair or via tear completion with subsequent repair) and debridement. To enhance comparability, all non-surgical treatment options are grouped into a single category.

While the choice of treatment is critical, most existing meta-analyses have focused on full-thickness tears [12, 13]. However, partial tears warrant equal consideration. Although some randomized controlled clinical trials (RCTs) [14] and meta-analyses [15] address partial tears, a standardized treatment algorithm for symptomatic is lacking [16]. Moreover, no meta-analysis to date has comprehensively synthesized the available evidence.

This systematic review and meta-analysis aim to evaluate the best available clinical evidence on surgical and non-surgical treatments for PT-RCTs, to compare their outcomes at the level of treatment strategies, and to provide a more detailed analysis of different surgical techniques.

## Methods

The protocol of this systematic review was prospectively registered in PROSPERO and follows PRISMA 2020 (Preferred Reporting Items for Systematic Reviews and Meta-Analyses), PRISMA-S and TARCiS guidelines [17–19]. 

### Inclusion and exclusion criteria

Only English-language studies were ultimately included; German texts were excluded based on other criteria. Eligible studies involved adults (≥ 18 years) with a confirmed PT-RCT via clinical assessment or imaging (MRI, arthrogram, or ultrasound). Only clinical studies reporting outcomes of surgical and/or non-surgical interventions were considered eligible, including both comparative and single-arm studies. A direct comparison between surgical and non-surgical treatments was not required, as pooled outcomes across treatment strategies were analyzed. Studies were excluded if they did not meet the inclusion criteria, met any exclusion criteria or if they involved asymptomatic individuals, animals, in vitro models, and cadaveric specimens.

**Fig. 1:**
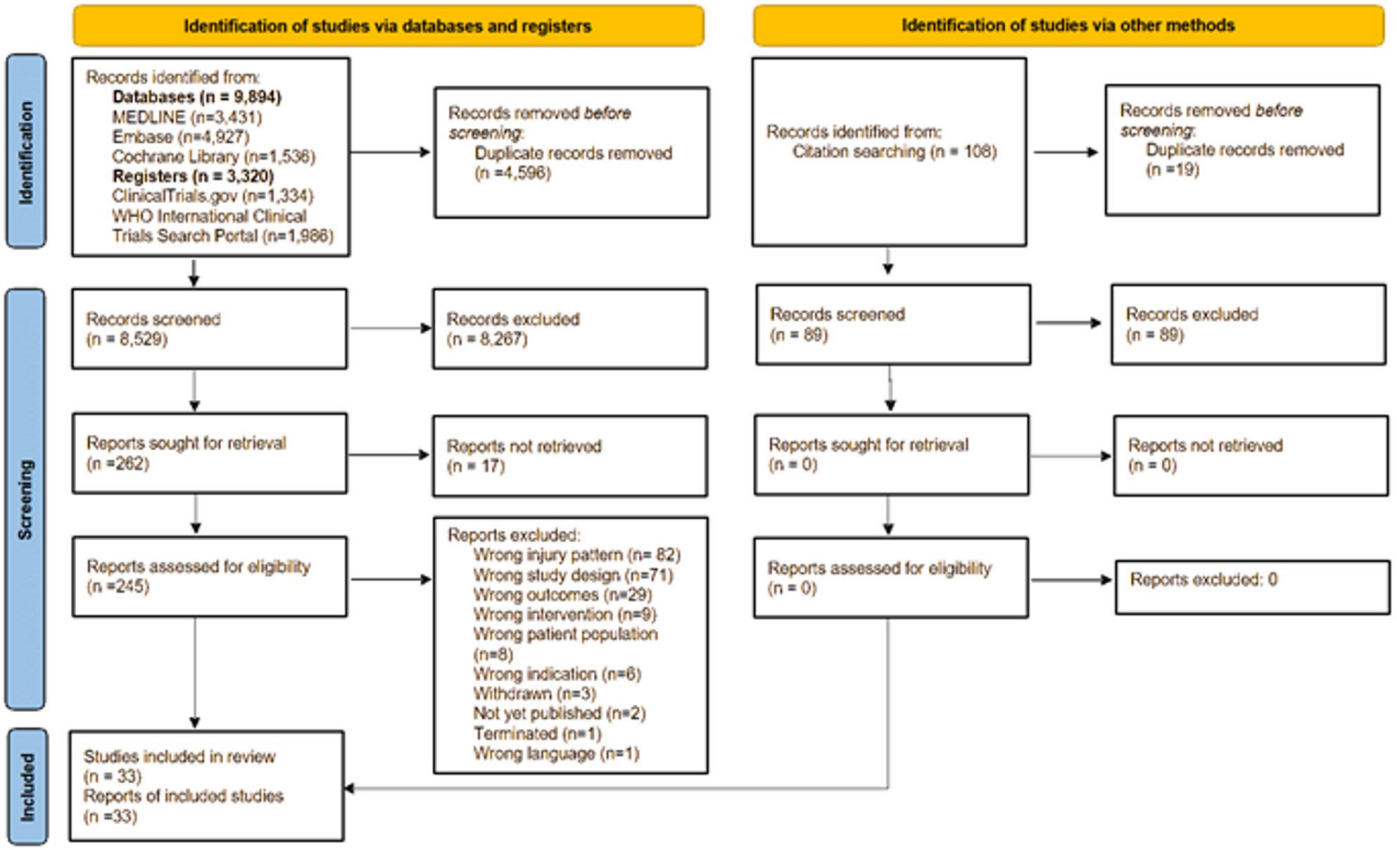
PRISMA Flow Diagram [17]

Reasons for full-text exclusions are detailed in the PRISMA flow diagram (Fig. 1).

### Study identification

Databases and registers were searched in January 2024 [20, 21]: MEDLINE (via Ovid), EMBASE (via Ovid), Cochrane Central Register of Controlled Trials (CENTRAL) (via Cochrane Library), Clinicaltrials.gov, World Health Organization (WHO) International Clinical Trials Registry Platform (ICTRP) (http://apps.who.int/trialsearch/). Search strategies for MEDLINE and Embase were adapted from recent Cochrane reviews and modified for other databases. The search focused on “Population: Partial rotator cuff tears” without filters for intervention, date or language. To provide a comprehensive synthesis of the available evidence on surgical and non-surgical treatment strategies for partial rotator cuff tears, the search strategy was designed to include studies reporting outcomes of either intervention independently. This approach enabled the inclusion of a broader range of studies and facilitated an indirect comparison between treatment modalities.

For the purpose of comparing overall treatment strategies, non-surgical interventions were considered as a single analytical category. Given the wide variability of non-surgical approaches reported in the literature, this strategy ensured a comprehensive inclusion of all eligible interventions without restriction to specific modalities. This approach was chosen to avoid selective inclusion and potential selection bias. The implications of this grouping, particularly with regard to clinical heterogeneity, were considered in the interpretation of the results. Backward citation searches were conducted via Lens.org and the citationchaser Shiny app [22] with manual check for completeness. Full search strategies and checklists are available in the public repository (see Data Availability Statement).

All search results were imported into Endnote (version 20.6) and deduplicated using the method of Bramer et al. [23] (steps A-C) and database accession numbers. Remaining records were then imported into Covidence [24] for further deduplication. Citation search results were imported directly into Covidence.

### Screening and data collection

Study screening and data collection were conducted using Covidence software. Two investigators independently screened titles, abstracts and full texts. To determine eligibility for each synthesis, the characteristics of each study’s intervention were extracted and compared with the predefined criteria to ensure alignment with the planned groups. Discrepancies were resolved through discussion with a third reviewer until consensus was reached. The included studies were analyzed by these two authors, who collected data on general study information, including study type, level of evidence, patient demographics, type of intervention, treatments, and outcomes. From studies that reported data on complete and partial ruptures, only the data on partial ruptures were extracted and used for this study. A key objective was to undertake a detailed examination of the individual interventions within the studies, with a view to facilitating comparisons between the different therapies. For each study, the outcomes from the final follow-up assessment were used for analysis, with the CS, ASES, and VAS serving as assessment tools. Six studies (Chun et al. [25], Godek et al. [26], Hurd et al. [27], Jo et al. [28], Kim, Y.S et al. [29], Lundeen et al. [30]) required the outcome values to be extracted from charts, whereas all other studies reported the outcome values directly in the text or tables.

### Data analysis

Statistical analyses were performed using R [31] (RStudio Version 2024.09.1 + 394) with the ‘metafor’ and ‘flextable’ packages. Two approaches were used for effect size estimation: Weighted mean (WM) was used as an absolute parameter to compare the overall surgical and non-surgical groups. Mean difference (MD) was applied only within surgical subgroups, where two groups could be directly compared for detailed analysis.

For MD- and WM-based analyses, statistical methods were selected according to the type of data and analysis performed. For WM-based analyses, study-level weighted means were pooled within surgical and non-surgical groups using a random-effects model to account for heterogeneity (assessed via I², τ², and χ²), with χ² and corresponding p-values used for subgroup comparisons. For MD-based analyses within the surgical group, mean differences were calculated where direct comparisons were available, with heterogeneity assessed using I², τ², and p-values, and the overall effect evaluated using the Z-value and corresponding p-value.

Heterogeneity was interpreted using the I² statistic, with the following thresholds: 0%–40% indicating low, 30%–60% moderate, 50%–90% substantial, and 75%–100% considerable heterogeneity. Forest plots were created to visualize heterogeneity and to present summary estimates across studies. In cases where substantial or considerable heterogeneity was detected, a random effects model was applied to account for variability between studies and to pool the results. Statistical significance was defined as a p-value below 0.05. When data was insufficient to conduct a meta-analysis, descriptive statistics were used to summarize and report outcomes. The impact of heterogeneity on the interpretation of pooled estimates was considered in the overall analysis.

## Results

### Study selection

**Fig. 2:**
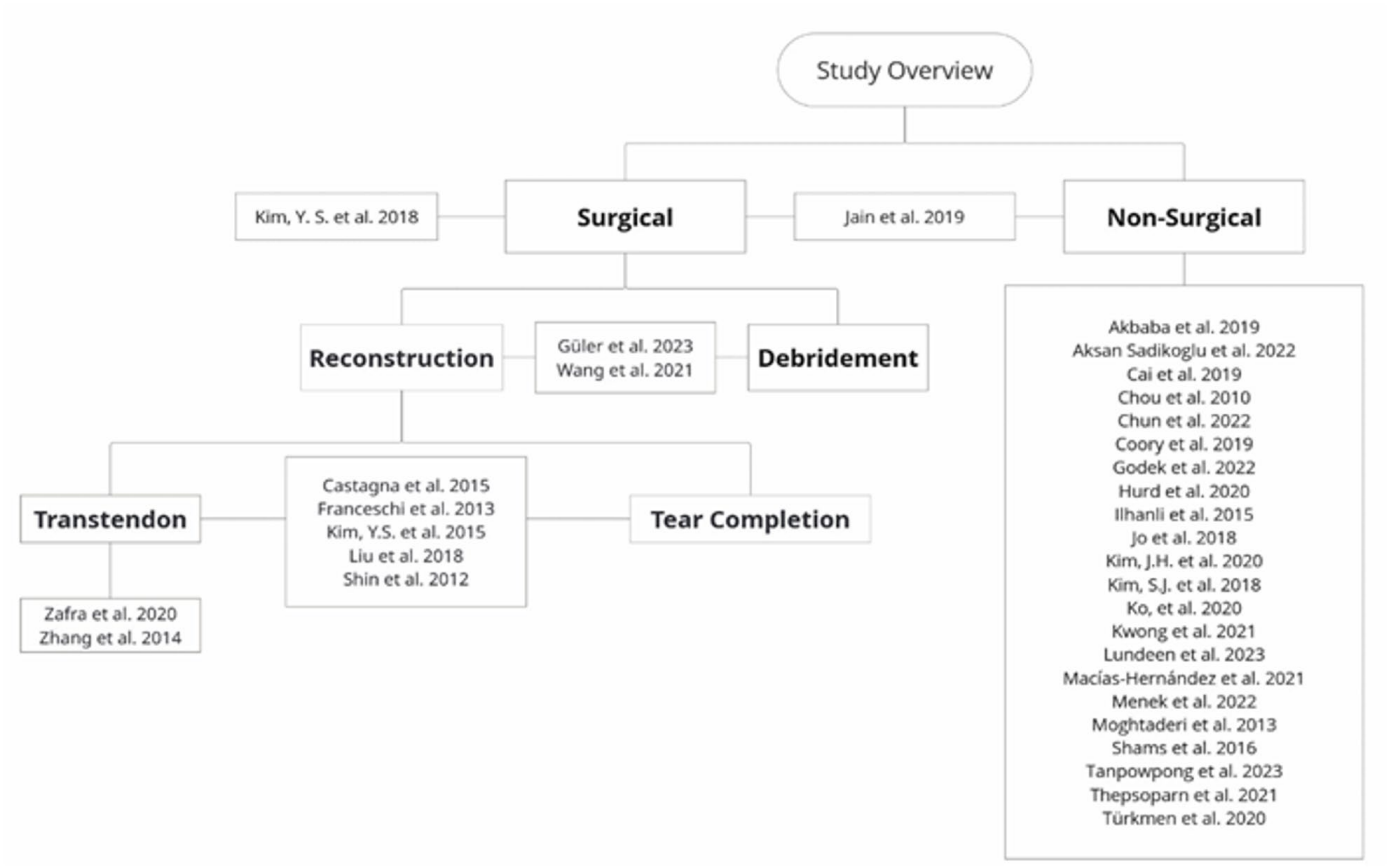
Study Overview

The database searches yielded 14,490 records. Following the removal of duplicates, 9,894 studies were retained for title and abstract screening. Of these, 245 studies were deemed pertinent and underwent further scrutiny at the full-text level. Of the 245 studies identified, 212 were excluded as they failed to meet the pre-established eligibility criteria, for reasons outlined in Fig. 1. Furthermore, a citation search was conducted, but none of the 89 additional records identified in this search was found to be eligible. We contacted the investigators or study sponsors only when necessary, such as when key study details required confirmation or numerical outcome data were missing, with a 14-day interval between each contact. This was particularly relevant for studies available only as abstracts or those lacking complete participant data. Despite these efforts, 17 studies could not be included in the final analysis due to the unavailability of the requisite information. Ultimately, 33 studies with a total of 1,818 patients were deemed to meet all the requisite criteria and were thus included in the review (Fig. 2). A direct comparison between surgical and non-surgical treatment was available in only one study, while the remaining studies reported outcomes for either surgical or non-surgical interventions independently.

### Arthroscopy vs. non-surgical

A Level IIc [32] study involving 127 patients was conducted to evaluate the efficacy of surgical and non-surgical treatments for rotator cuff ruptures [33]. Of these, 50 patients underwent arthroscopic treatment, while 77 were managed non-surgically. The study was included in the review because it explicitly separated patients with complete and partial ruptures (89.6% vs. 10.4% in the surgical group and 54.8% vs. 45.2% in the non-surgical therapy group). This clear distinction allowed us to extract and use only the data for partial-thickness ruptures in our analysis. The ASES Score was used to present the results, showing a statistically significant improvement in surgically treated patients compared to those managed non-surgically. In comparison to baseline data, 88% of surgical patients showed > 30% improvement versus 61% of non-surgical patients (*p* = 0.002), while 86% of surgical patients demonstrated > 50% improvement, compared to 44% in the non-surgical group (*p* < 0.0001).

### Arthroscopy

There was one RCT with a total of 78 patients. The Kim, Y. S et al. [34] study classified as Level II evidence, recorded CS, ASES Score, and VAS, presenting the results in a line chart comparing immediate arthroscopic rotator cuff repair to repair after six months of non-surgical therapy. Both groups showed statistically significant improvements in all three scores when post-operative results were compared with baseline data. Although the non-surgically pretreated group had significantly higher ASES and VAS scores at the six-month follow-up, this difference was no longer present at the twelve-month follow-up.

### Debridement vs. reconstruction

There were two studies with a total of 125 patients. Of the total number of patients, 59 were assigned to the debridement group, while 66 were allocated to the reconstruction group. The study by Wang et al. [35] provides Level II evidence, whereas the study by Güler et al. [36] is classified as Level III evidence. Both studies report their results using the CS, the ASES Score, and the VAS, with a follow-up period of 18 months.

The results of Güler’s study show a significant improvement in all scores within each group between the pre- and postoperative assessments (*p* = 0.01) for all scores. Postoperatively, a significant difference was observed between the groups, with the reconstruction group showing greater improvement compared to the debridement group (CS and ASES, *p* = 0.001; VAS, *p* = 0.008). Similarly, Wang’s study demonstrated a significant pre- to postoperative improvement within each group (*p* < 0.0001). However, the comparison between groups did not reach statistical significance (CS, *p* = 0.664; ASES, *p* = 0.301; VAS, *p* = 0.463).

### Transtendon vs. tear completion

There were five studies with a total of 342 patients. Of these, 170 patients were assigned to the transtendon group, while 172 were allocated to the tear completion group. Four studies are RCTs and thus classified as Level II evidence, while one study (Liu et al. [37]) is Level III. Among the five studies, three (Franceschi et al. [38], Kim et al. [34], Shin et al. [39]) reported their results using both, CS and ASES Score. In contrast, one study (Castagna et al. [40]) used solely CS, while another (Liu et al.) used only ASES. Furthermore, four studies (Castagna et al., Kim et al., Liu et al., Shin et al.) included VAS as an outcome indicator.

Two additional studies focusing exclusively on transtendinous repair (Zafra et al. [41], Zhang et al. [42]) were also included, comprising a total of 85 patients. Zafra presented the results using all three scores, whereas Zhang solely reported ASES, which increased from a baseline of 48.34 to 87.88 after 24 months. However, neither study demonstrated statistical significance.

All studies recorded outcomes with a follow-up period of at least six months, with the longest follow-up extending to three years, as observed in Franceschi et al. and Zafra et al. The evaluation of outcome indicators across all studies revealed a statistically significant postoperative improvement compared to the preoperative conditions within each group (*p* ≤ 0.001). However, when the groups were compared with one another, no statistically significant differences were observed (*p* = 0.06 to *p* = 0.97).

### Non- surgical

A total of 22 studies involving 1,146 patients were included. Among them, twenty studies are RCTs and therefore provide Level II evidence, while one study (Kim, S.J. et al. [43]) is classified as Level III evidence, and one study (Jo et al. [28]) as Level IV evidence. For the purposes of this study, all 22 studies focusing on non-surgical therapy have been grouped into a single category to enable comparison with surgical treatment approaches. The included studies covered a broad range of interventions, including injections (both cell-based and non- cell-based options), physical therapy (eccentric, concentric, MTrPS, etc.) and a comparison between both approaches. This grouping introduces substantial clinical heterogeneity and may obscure differences between individual non-surgical interventions.

Kim, J.H. et al. [44] and Cai et al. [45] present their findings using all three outcome parameters (CS, ASES Score, VAS). Cai reports a superiority of Platelet-Rich Plasma (PRP) injections (*p* < 0.05), while Kim found that a statistically significant difference (*p* ≤ 0.01) between patients treated with Atelocollagen (*n* = 76) and those who did not receive Atelocollagen (*n* = 38).

Among the studies that assessed both, ASES and VAS (Akbaba et al. [46], Aksan Sadigoklu et al. [47], Chun et al. [25], Hurd et al. [27], Kim, S.J. et al. [43], Kwong et al. [48], Lundeen et al. [30], Türkmen et al. [49]), Hurd reported the superiority of UA-ADRCs (*p* < 0.05), while Kwong found PRP to be superior to Corticosteroid (CSI) (*p* = 0.03). In Kim, S.J.’s study, a statistically significant improvement in the bone marrow therapy group after three months compared to the manual therapy group was observed (ASES, *p* = 0.011; VAS: *p* = 0.039). The remaining studies did not yield statistically significant results.

Of the studies that employed both, CS and VAS measurements (Chou et al. [50], Coory et al. [51], Jo et al. [28], Ko et al. [52], Macias-Hernandez et al. [53], Moghtaderi et al. [54]), Macias Hernandez was the only study that did not report significant findings between groups. Chou found no significant differences during the treatment period, but at the six-week follow-up, a significant improvement in VAS was observed in the sodium hyaluronate compared to placebo (*p* = 0.002), indicating the superiority of sodium hyaluronate, though no such difference was seen in the CS group (*p* = 0.091). Coory reported a statistically significant improvement in outcomes with nerve block compared to subacromial injection (*p* = 0.03), while Jo’s findings indicate that a higher dose of Adipose-Derived Mesenchymal Stem Cells (AD MSCs) was associated with significant improvements in outcome scores (*p* < 0.001). In comparison of extracorporeal shock wave therapy with sham therapy, Ko reported a significant improvement in CS (*p* = 0.005) and VAS (*p* = 0.025).

Moghtaderi’s results suggested that sodium hyaluronate was more effective than placebo (*p* < 0.001).

Both, Shams et al. [55] and Tanpowpong et al. [56], found PRP to be superior to CSI at the 12 week follow-up based on CS and ASES outcomes. In Shams et al., the differences were highly significant (*p* < 0.001 for both CS and ASES), while in Tanpowpong et al., significance was also observed (CS: *p* = 0.02, ASES: *p* = 0.002). Godek et al. [26], Ilhanli et al. [57], Menek et al. [58], and Thepsoparn et al. [59] exclusively used VAS as an outcome measure. Of these studies, Ilhanli reported a notable improvement in VAS for rest (*p* = 0.045), whereas no significant changes were observed in VAS for sleep or activity. Thepsoparn identified a statistically significant result, showing that PRP led to superior outcomes compared to CSI (*p* < 0.01) at the six-month follow-up.

### Retear as main complication

Of the ten studies on surgical treatment, five (Kim [34], Kim [29], Shin [39], Wang [35], Zafra [41]) consider the retear rate as a distinct outcome parameter.

A more detailed evaluation of retear rates revealed that most studies assessed structural integrity using postoperative imaging based on the Sugaya classification [60]. In the study by Kim [34] three retears were detected by MRI at 12 months, with one case in the immediate repair group (*n* = 44) and two cases in the delayed repair group (*n* = 34). No statistically significant difference between groups was observed. Similarly, Kim [29] reported two retears in the transtendon repair group (*n* = 47) and seven in the tear completion group (*n* = 45). While no significant difference was found for articular-sided tears, a significantly higher retear rate was observed in bursal-sided tears treated with tear completion (*p* = 0.02). In line with these findings, Shin [39] also reported retears exclusively in the tear completion group, with two cases identified (*n* = 24), whereas no retears were observed in the transtendon group.

Wang [35] reported no retears in their cohort. Zafra [41] observed two retears in the single-row group (*n* = 25) and one in the double-row group (*n* = 25), with no statistically significant difference between groups.

Two studies (Güler [36], Liu [37]) only address it in the discussion section. Three studies (Castagna [40], Franceschi [14], Jain [33]) do not report this outcome at all. Overall, retear rates across studies were low (6.3%); however, the small number of events and the heterogeneity in surgical techniques, tear types, and reporting methods limit the ability to draw robust comparative conclusions.

### Methodological quality assessment

**Fig. 3:**
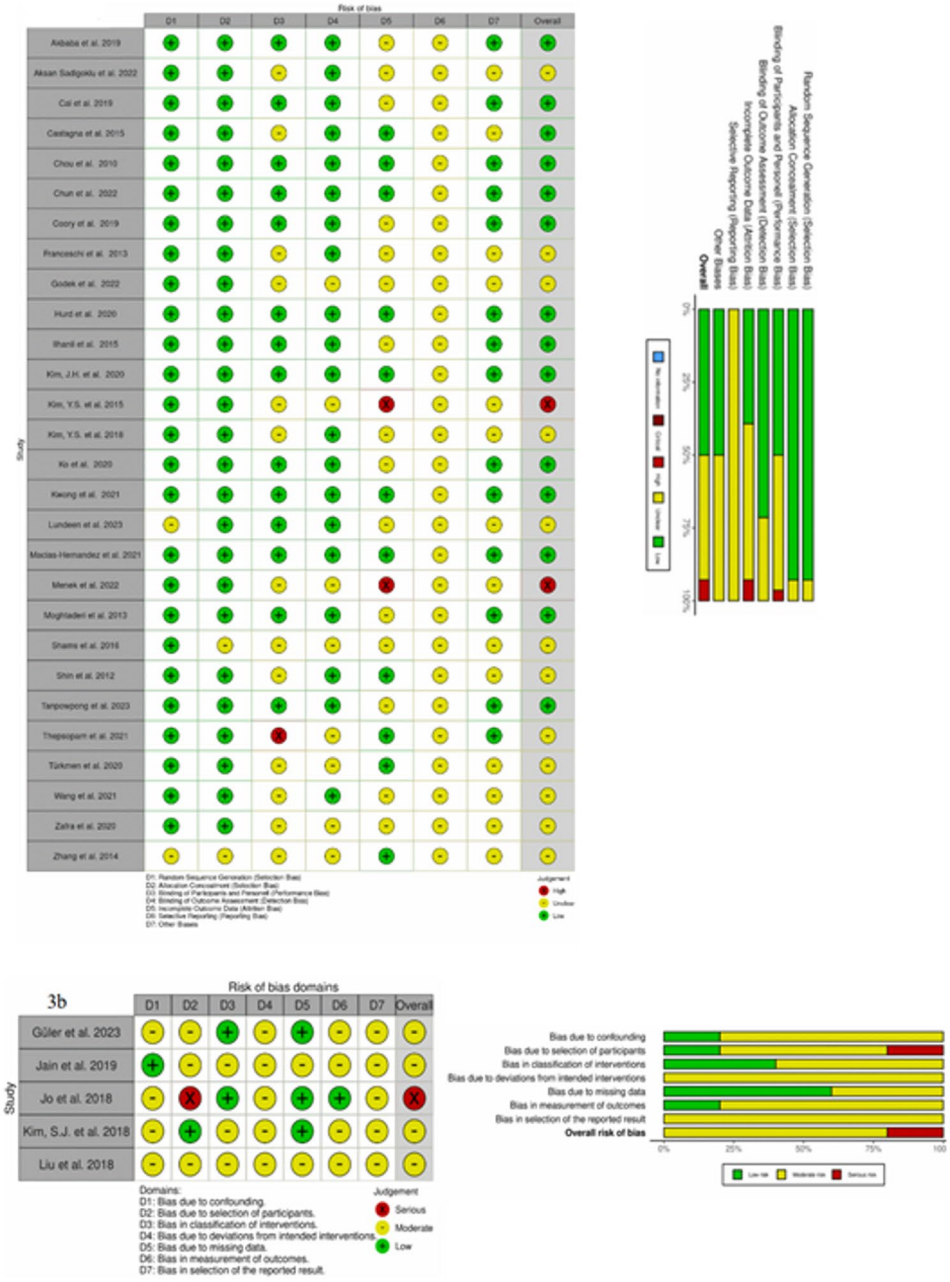
3**a** Cochrane risk of bias RoB2 (RobGeneric) tool for randomized studies; 3**b** Cochrane risk of bias ROBINS-I tool for non-randomized studies [61]

Bias was assessed using the Cochrane Risk of Bias Tool for RCTs and non-randomized studies. Of the 33 included studies, 28 were RCTs with a high level of randomization, reducing selection bias (Fig. 3a). Performance and selection bias were generally low to moderate due to frequent blinding. Attrition and detection bias were well controlled, but reporting bias remained a notable concern. Overall, evidence quality was mixed, ranging from well-controlled to high-risk studies. Of particular relevance is that the varying evidence levels across the included studies contribute to a differential risk of bias.

### Meta- analysis

**Fig. 4:**
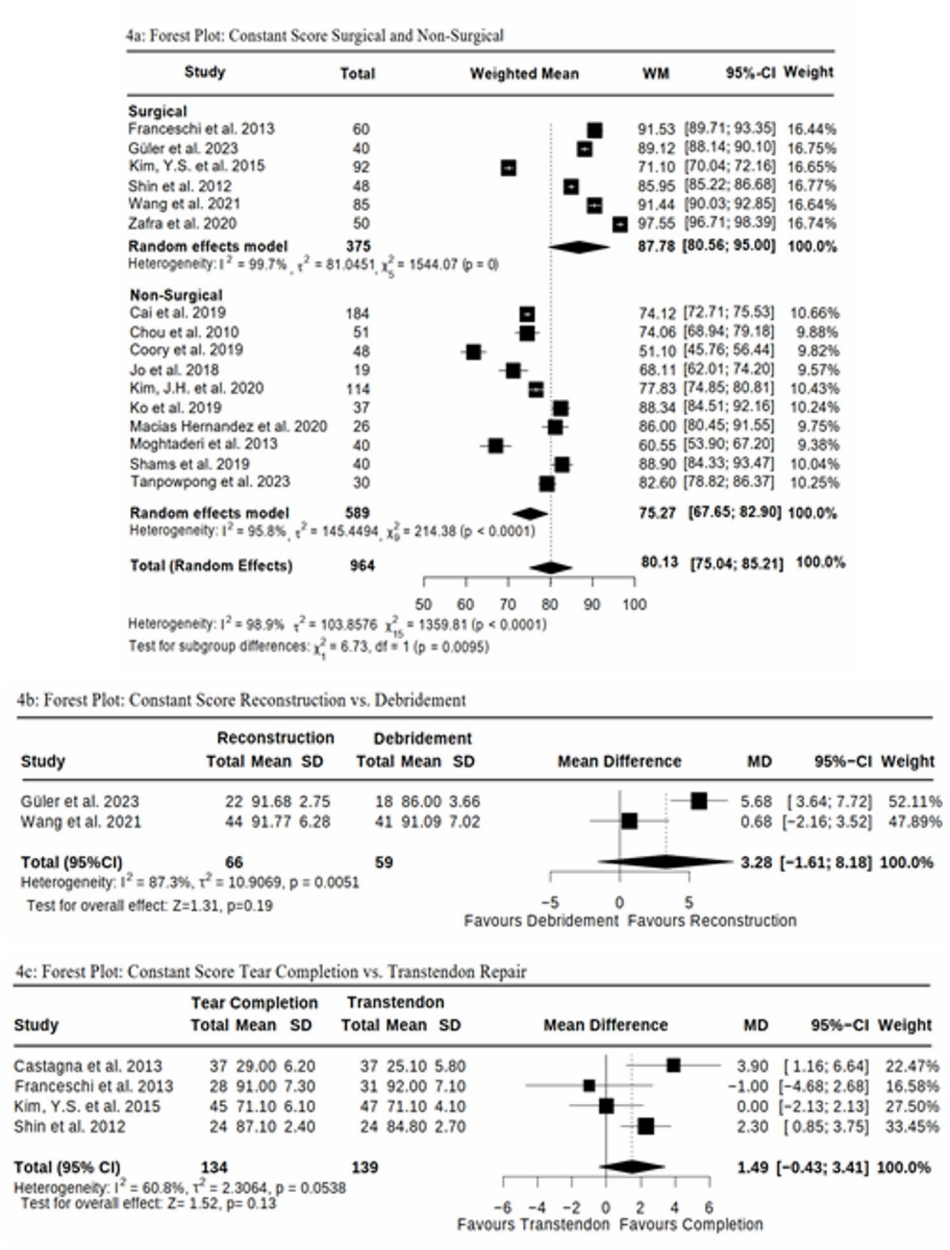
4**a**-**c** Forest Plots for Constant Score (CS) between; 4**a** Surgical vs Non-surgical Interventions; 4**b** Surgical Reconstruction vs Debridement; 4**c** Surgical Tear Completion vs. Transtendon Repair

**Fig. 5:**
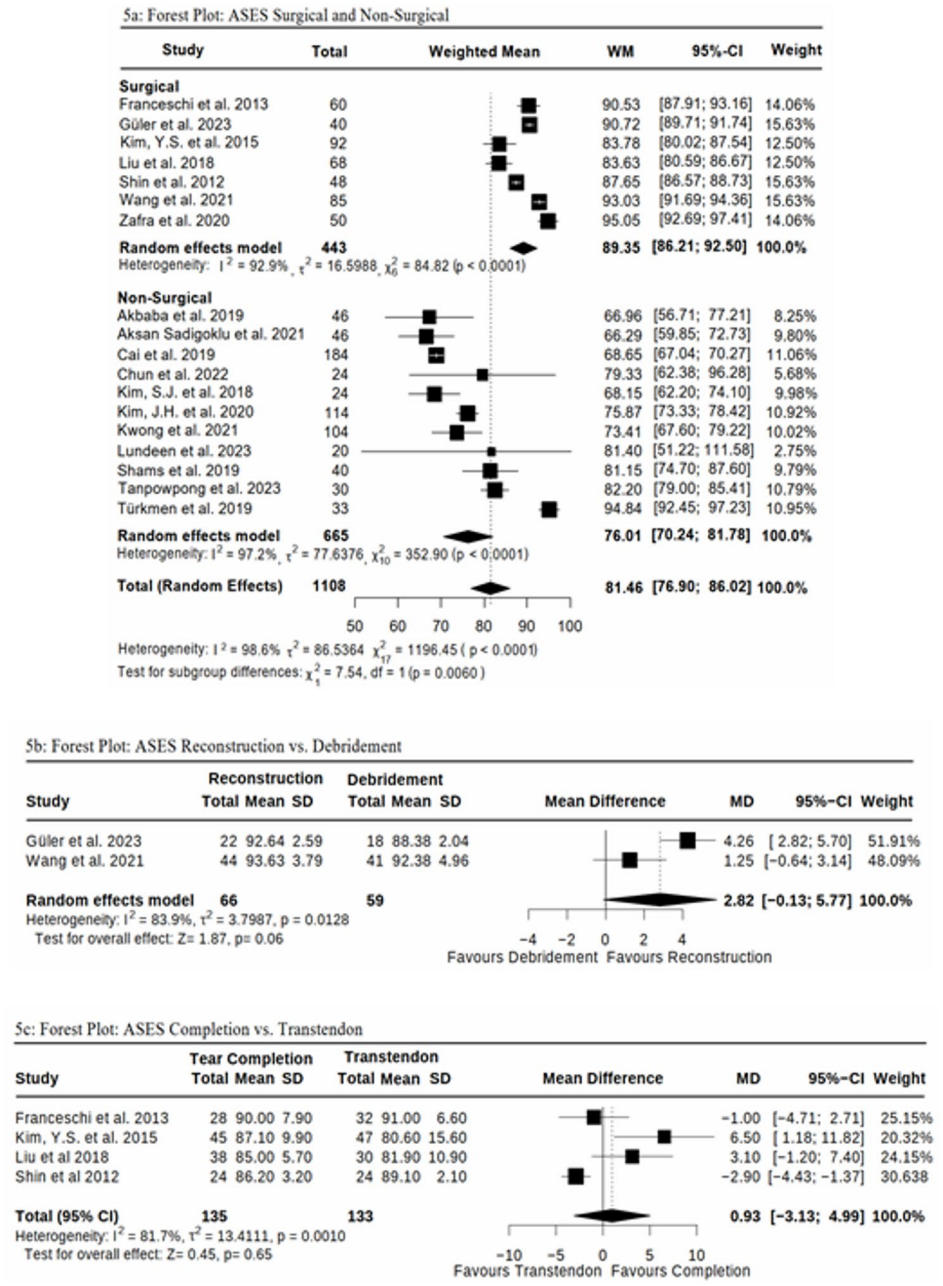
5**a**-**c** Forest Plots for ASES between: 5**a** Surgical vs Non-surgical Interventions; 5**b** Surgical Reconstruction vs Debridement; 5**c** Surgical Tear Completion vs. Transtendon Repair

Due to methodological differences and variations in the outcome measures across the available studies, only 25 studies were included for comparison. Hurd et al. [27] and Kim, Y.S. et al. [34] could not be included due to missing standard deviation values. It was not possible to include Zhang et al. [42] due to the lack of a suitable comparison group. Since our focus was on the statistical analysis of the ASES and CS, Godek et al. [26], Ilhanli et al. [57], Menek et al. [58], and Thepsoparn et al. [59] were excluded from the statistical evaluation, as they focused solely on VAS (Figs. 4 and 5).

## Discussion

When comparing pooled weighted mean outcomes across studies, surgical cohorts demonstrated higher scores than non-surgical cohorts in both the CS (*p* = 0.0095, Fig. 4a) and the ASES (*p* = 0.0060, Fig. 5a). However, given that these findings are based on indirect comparisons between separate study populations, they should be interpreted with caution and do not allow for causal inference regarding the superiority of surgical treatment. 

A detailed analysis of the mean differences between surgical interventions reveals a statistically non-significant improvement in Reconstruction compared to Debridement, as indicated by the CS (p = 0.19, 95%-CI (-1.61; 8.18), Fig. 4b) and the ASES (p = 0.06, 95%-CI (-0.13;5.77), Fig. 5b). Similarly, although Tear Completion appears to show a numerical advantage over Transtendon Repair, this difference remains non-significant for both the ASES (p = 0.65, 95%-CI (-3.13; 4.99), Fig. 5c) and the CS (p = 0.13, 95%-CI (-0.43; 3.41), Fig. 4c). All reported 95% confidence intervals include zero, suggesting that the differences are modest and do not provide strong evidence favoring one technique over another.

Although some results demonstrate statistical significance, the findings remain below the minimal clinically important differences (MCID) of 10.4 points for the CS [62] and below the 11–17-point range for the ASES [56, 63] MCID. Importantly, the evidence for subgroup analyses comparing different surgical techniques is limited, with only a small number of studies available. As a result, these analyses are underpowered, and the clinical significance of observed differences is restricted. The findings should therefore be interpreted with caution.

This review and meta-analysis are among the first to comprehensively evaluate surgical and non-surgical treatment approaches specifically for PT-RCTs. To broaden the clinical context, findings from studies on FT-RCTs were also considered. While Ryösä et al. [12] and Longo et al. [13] observed a trend favoring surgery over non-surgical care, results were not statistically significant. In contrast, Piper et al. [64] and Schemitsch et al. [65] reported statistically significant benefits for surgery, though differences were not clinically meaningful. These findings are consistent with our results for PT-RCTs.

Eubank et al. [10] concluded that both treatment modalities improve pain and function in PT-RCTs, without evidence favoring one over the other. The authors recommend surgery primarily for cases unresponsive to appropriate non-surgical measures, a concept that is supported by recent evidence suggesting that conservative treatment may be insufficient in patients with high-grade (> 50%) bursal-sided tears, particularly when severe pain limits tolerance, making early surgical intervention a reasonable option in this subgroup [66]. However, this does not diminish the importance of an integrated treatment approach, in which physical therapy remains essential – both as in initial non-surgical management [67] and as part of post-operative and post-traumatic rehabilitation [68]. Overall, differences between specific conservative techniques appear to be minor, with, for example, one study reporting similar outcomes between landmark- and ultrasound-guided injections, underscoring the greater importance of treatment strategy and patient selection [69]. 

Regarding surgical techniques, Goyal et al. [70] found comparable outcomes between trans-tendon repair and tear completion in terms of CS and ASES, which aligns with our functional outcome data. This is further supported by a recent meta-analysis demonstrating no significant differences between in situ repair and tear completion in the management of bursal-sided PT-RCTs with respect to functional outcomes, pain scores, or retear rates [71]. However, Goyal emphasizes lower retear rates with trans-tendon repair. Given retear risks of 5–10% in small to medium tears and up to 50% in large or massive tears, this is clinically relevant. Factors such as patient age, tendon degeneration and muscle atrophy, and comorbidities like diabetes, smoking and osteoporosis can further elevate this risk. Persistent postoperative pain and/or weakness may be indicative of a retear [72]. 

Partial-thickness tears are typically classified according to Ellman’s system, which differentiates tears based on their location - articular, bursal, or intra-tendinous - and depth: less than 3 mm (< 25%), 3–6 mm (25–50%), and greater than 6 mm (> 50%) [73]. This classification is clinically relevant, as studies have demonstrated that the symptom severity often increases with tear size, and even small asymptomatic tears can progress and become irreparable over time if left untreated [74]. 

Considering the limitations, several key methodological aspects should be acknowledged.

The overall study design is largely based on indirect comparisons across separate study populations. While this approach allowed us to address the clinically relevant question of surgical versus non-surgical treatment strategies, it does not permit causal inference and limits the strength and certainty of the conclusions. Direct comparative evidence between surgical and non-surgical management is limited, and the main analyses of this review are therefore based on pooled comparisons across separate study cohorts. The results should be interpreted within this framework.

In addition, all non-surgical interventions were grouped into a single analytical category. Given the broad range of non-surgical treatment modalities reported in the literature, this approach was chosen to enable comparison at the level of overall treatment strategies and to avoid selective inclusion of specific interventions. However, non-surgical treatment strategies are inherently heterogeneous, encompassing a wide range of modalities with different mechanisms of action and treatment intensities. This grouping therefore introduces substantial clinical heterogeneity and may limit the interpretability of pooled estimates for individual non-surgical treatments.

Furthermore, considerable methodological and clinical heterogeneity was present across the included studies, arising from differences in study design, patient populations, tear characteristics, and treatment approaches. In particular, studies varied in follow-up duration, patient demographics, and reporting of tear type and severity, with inconsistent differentiation between articular- and bursal-sided tears. In many cases, the extent of tendon involvement was not uniformly reported, further limiting comparability between study populations. Consequently, differences in patient selection, treatment approaches, and outcome reporting increase uncertainty in the pooled estimates. Greater standardization in the reporting of partial-thickness rotator cuff tears would improve comparability across studies and enable more detailed analyses of specific tear types, thereby facilitating more meaningful pooling of results and supporting both scientific evaluation and clinical decision-making.

Additional sources of heterogeneity include variation in patient age, activity level, and surgical techniques. These factors limit direct comparability, reduce the precision of pooled estimates, and affect the generalizability of the findings, which is further reflected in the substantial statistical heterogeneity observed (high I² values). In addition, no sensitivity analyses restricted to higher-quality studies were conducted, further limiting the ability to assess the robustness of the findings. Therefore, these limitations should be considered when interpreting the pooled results.

Another key limitation is that, although the majority of the included studies are RCTs (level II), five studies were of lower evidence levels (level III or IV), increasing the risk of bias and potentially impacting the reliability of the overall results.

Nevertheless, this meta-analysis has several important strengths: Most notably, the comprehensive search strategy and the fact that over 80% of the studies included are RCTs provide a solid evidence base. Moreover, by considering three distinct outcome parameters, this analysis allows for a thorough comparison across studies. The integration of diverse studies using similar therapeutic approaches enables a robust meta-analytic evaluation, allowing not only a general comparison between surgical and non-surgical treatments but also more differentiated comparisons within these groups.

In conclusion, despite inherent limitations that should be considered when interpreting the findings, this meta-analysis offers a valuable and comprehensive synthesis of the current evidence regarding the management of PT-RCTs. Nevertheless, larger studies with longer follow-up periods are needed to allow for a more precise assessment of treatment outcomes and long-term effects.

## Conclusion

This systematic review and meta-analysis are among the first to provide a comprehensive synthesis of the available evidence on surgical and non-surgical treatments for PT-RCTs. While pooled analyses showed higher Constant and ASES scores in surgical cohorts, these differences remained below the MCID, indicating a lack of clinically meaningful benefit. Given the reliance on indirect comparisons, these findings should be interpreted with caution and do not support causal conclusions regarding the superiority of surgical treatment. No surgical technique demonstrated clear superiority.

Overall, the findings support individualized, patient-centered treatment decisions that consider both surgical and non-surgical options and do not support a clinically meaningful superiority of surgical over non-surgical treatment. Further high-quality randomized controlled trials with direct comparisons are required to establish optimal treatment strategies.

## Data Availability

Data supporting this study is available from a public repository: 10.5283/epub.79413. This includes search strategies, accession numbers of the records found and PRISMA-S and TARCiS checklists for the search process, PRISMA 2020 checklist, list of excluded studies and a table of included studies.

## Abbreviations

AD-MSC: Adipose-Derived Mesenchymal Stem Cells
ASES: American Shoulder and Elbow Surgeons Shoulder Score
CS: Constant Score
CSI: Corticosteroid Injection
FT-RCT: Full-thickness Rotator Cuff Tears
ICTRP: International Clinical Trials Registry Platform
MCID: Minimal Clinically Important Differences
MD: Mean Difference
MRI: Magnetic Resonance Imaging
MTrPs: Myofascial Trigger Points
PRISMA: Preferred Reporting Items for Systematic Reviews and Meta-Analyses
PRP: Platelet Rich Plasma
PT-RCT: Partial-thickness Rotator Cuff Tears
RCD: Rotator Cuff Disease
RCT: Randomized Controlled Trial
UA-ADRCs: Uncultured, Unmodified, Autologous Adipose-derived Regenerative Cells
VAS: Visual Analogue Scale
WHO: World Health Organization
WM: Weighted Mean
